# COVID-19 one year into the pandemic: from genetics and genomics to therapy, vaccination, and policy

**DOI:** 10.1186/s40246-021-00326-3

**Published:** 2021-05-10

**Authors:** Giuseppe Novelli, Michela Biancolella, Ruty Mehrian-Shai, Vito Luigi Colona, Anderson F. Brito, Nathan D. Grubaugh, Vasilis Vasiliou, Lucio Luzzatto, Juergen K. V. Reichardt

**Affiliations:** 1 Department of Biomedicine and Prevention, “Tor Vergata” University of Rome, 00133 Rome, Italy; 2 IRCCS Neuromed, Pozzilli, IS Italy; 3 Department of Pharmacology, School of Medicine, University of Nevada, Reno, NV 89557 USA; 4 Department of Biology, Tor Vergata University of Rome, 00133 Rome, Italy; 5 Pediatric Hemato-Oncology, Sheba Medical Center, Tel Hashomer, Israel; 6 Department of Epidemiology of Microbial Diseases, Yale School of Public Health, New Haven, CT 06510 USA; 7 Department of Environmental Health Sciences, Yale School of Public Health, New Haven, CT 06510 USA; 8 Haematology, Muhimbili University of Health and Allied Sciences, Dar-es Salaam, Tanzania; 9 Australian Institute of Tropical Health and Medicine, James Cook University, Smithfield, Queensland 4878 Australia

**Keywords:** Coronavirus, SARS-CoV-2, COVID-19, Pandemic, Variants, Vaccines, Monoclonal antibodies, Politics

## Abstract

COVID-19 has engulfed the world and it will accompany us all for some time to come. Here, we review the current state at the milestone of 1 year into the pandemic, as declared by the WHO (World Health Organization). We review several aspects of the on-going pandemic, focusing first on two major topics: viral variants and the human genetic susceptibility to disease severity. We then consider recent and exciting new developments in therapeutics, such as monoclonal antibodies, and in prevention strategies, such as vaccines. We also briefly discuss how advances in basic science and in biotechnology, under the threat of a worldwide emergency, have accelerated to an unprecedented degree of the transition from the laboratory to clinical applications. While every day we acquire more and more tools to deal with the on-going pandemic, we are aware that the path will be arduous and it will require all of us being community-minded. In this respect, we lament past delays in timely full investigations, and we call for bypassing local politics in the interest of humankind on all continents.

**Supplementary Information:**

The online version contains supplementary material available at 10.1186/s40246-021-00326-3.

## Introduction

The coronavirus disease 2019 (COVID-19), caused at individual level by the severe acute respiratory syndrome coronavirus 2 (SARS-CoV-2), has raged for a year now, as declared by the WHO (World Health Organization) [1]. By March 31, 2021, more than 125 million cases of SARS-CoV-2 infection have been reported, causing 2,816,081 deaths in 192 countries (Johns Hopkins University, CSSE). About the Coronavirus Treatment Acceleration Program (CTAP), there are more than 590 drug development programs in planning stages (i.e., antivirals, immunomodulators, cell and gene therapies, compound combinations and other active principles, vaccines excluded), more than 430 clinical trials reviewed by FDA (Food and Drug Administration) with a total of 9 COVID-19 treatments currently approved for use under Emergency Use Authorization (EUA) (https://www.fda.gov/drugs/coronavirus-covid-19-drugs/coronavirus-treatment-acceleration-program-ctap, accessed on March 31, 2021) [2]. Due to socioeconomic inequalities, such clinical trials are mainly conducted in/by institutions in high income countries. Disparities also impose large limitations on low/mid income countries, which are often unable to cope with high demands for molecular testing, variant surveillance, vaccine distribution, or to address the needs of their citizens for financial aid, to survive amid tough epidemic control measures. This shows that the effects of this pandemic may well be far-reaching and long-lasting. The central role and responsibility of the UN (United Nations) and specifically of the WHO are paramount. The very word pandemic means that it cannot be confronted by measures that are only at the level of a region, or of a country, or even of a continent: global measures are absolutely needed.

## On the origin of the COVID-19 pandemic

We note that last year already the WHO set up a panel to investigate the origin, preparedness, and response to the COVID-19 pandemic (https://www.who.int/news/item/09-07-2020-independent-evaluation-of-global-covid-19-response-announced) [3]. Regrettably, there has been only limited progress and the origin of the pandemic has not been definitively pinned down (https://www.sciencemag.org/news/2021/03/compromise-who-report-resolves-little-pandemic-s-origins-details-probe-s-next-steps) [4]. All of us, whether directly affected patients or not, should be concerned by such astounding delays, since it is of immense common interest for all to be best prepared for the next pandemic that will inevitably befall us eventually. We lament these delays and we call for expeditious, effective, and scientifically rigorous action by the WHO and the UN. We are not suggesting that these delays are politically motivated: but we are anxious for these organizations to show clear evidence that they are driven at all times by their feeling of responsibility toward all humankind. As scientists who are also citizens from around the world, we wish to be able to say that they are now acting more nimbly and self-assuredly. We also wish to highlight the importance of being mindful that emerging nations that will need thoughtful assistance in order to face the immediate health crisis, as well as the economic recovery thereafter: they need improved and more resilient health systems on a medium to long-term basis, as well as food security. In this context, a critical role should be played by other international organizations such as the EU (European Union) and notably the FAO (Food and Agriculture Organization of the UN). However, other regional organizations, such as ASEAN (Organization of Southeast Asian States), the AU (African Union), and the OAS (Organization of American States), should also play a significant and culture-sensitive role in their respective geographic areas.

## Virus variants

An important reason of concern for all countries is the emergence of virus variants as a result of mutation(s) during the current pandemic. RNA viruses, such as SARS-CoV-2, despite being endowed with proofreading activity during viral replication [5], have a high mutation rate, and the absolute number of mutations increases with every round of infection [6]. The average evolutionary rate of SARS-CoV-2 is ~0.8 × 10^−4^ substitutions per site per year, which equals about 2 substitutions (“mutations”) per month. Mutations that are deleterious or even lethal to the virus will be purged from the population, and we do not need to worry about those. Many mutations are essentially neutral, and are maintained in the population: they may not readily promote functional changes, but they may facilitate adaptation upon changes in the environment explored by viruses [7, 8]. However, a few mutations may be beneficial to the virus [9] because they lead to (i) increased transmissibility; (ii) higher infectiousness, (iii) higher virulence entailing higher rate of severe disease; (iv) immune/vaccine escape; or (v) any combination of the above.

**Fig. 1:**
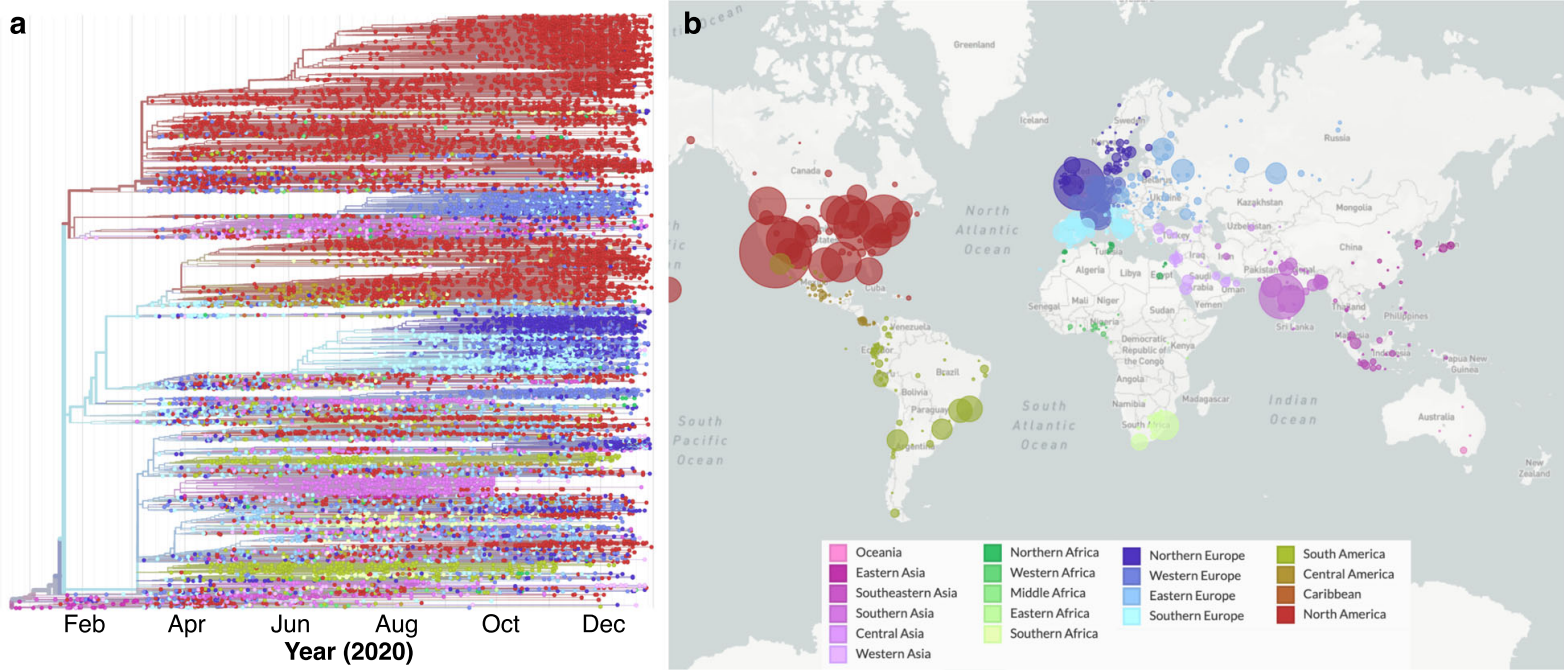
SARS-CoV-2 genomic surveillance. **a** Phylogenetic tree of 19,438 SARS-CoV-2 genomes. Up to December 31, 2020, more than 295,000 complete genomes were submitted to the GISAID database. Each circle in this tree represents a genome that was sequenced over time, since the beginning of the pandemic. As SARS-CoV-2 establishes new infections, its descendants form lineages of genetically related viruses, which can circulate more locally, as shown in lineages represented by threads of circles with similar colors (as shown in red, at the top of panel **a**), or may have more heterogeneous distributions, as depicted in lineages with multiple colors, highlighting the exchange of viruses between geographic regions (as shown at the bottom of panel **a**). **b** State level distribution of SARS-CoV-2 genomes shown in panel **a** (see acknowledgement Table S1). All regions of the world were impacted by the pandemic, some more than others. As a result, an imbalance in the distribution of genomes worldwide (depicted in panel **b** as bubbles of distinct sizes) is evident. The differences in genome sampling across continents and countries may not only be the result of epidemic control via distinct public health strategies (as observed in New Zealand and Australia, for example) but may also result from socioeconomic disparities at national and international scales, where some regions (e.g., South and Central America, most regions in Africa), despite being hard hit by the pandemic, are unable to conduct genomic surveillance at a scale comparable to that of rich countries in North America and Europe. Analyses and illustrations were respectively generated using augur and auspice (by nextstrain.org) [11]

Up to March 31, 2021, nearly 1 million (931,463) SARS-CoV-2 genomic sequences were submitted to the GISAID, the main database used by researchers in the field (https://www.gisaid.org) [10] (Fig. 1, Table S1). SARS-CoV-2 variants identified are heterogeneously distributed in geographic areas of the world (see https://nextstrain.org/ncov/global) [11, 12]. Variants of SARS-CoV-2 may adapt differently to the host under individual selective pressures [7]; some mutations may increase in frequency, either through genetic drift or through selection, and become fixed in different populations [6, 7]. This is the case of the mutation that led to the amino acid change D614G (Asp614 → Gly) in the spike glycoprotein (S), found in the predominant form of SARS-CoV-2 [13, 14]. Patients infected with the D614G variant often have higher viral loads in the upper respiratory tract than seen with the ancestral strain, but there seems to be no difference in disease severity [15]. The D614G mutation determines an important conformational change in the spike protein between the S1 and S2 domains, that favors the binding to the angiotensin-converting enzyme 2 (ACE2) receptor and thus increases the probability of infection: presumably the key to this variant having become globally dominant [15]. Recently, Huang et al. [13] have attributed the selective advantage of D614G variant to the quantitative differences in ACE2 expression in different populations. The lower ACE2 expression observed in the European, North American, and African populations, compared to Asians, may have driven positive selection favoring the D614G mutation.

Many SARS-CoV-2 mutations appeared and were selected for several times, independently, e.g., those that changes the asparagine residue at spike position 501 (S:N501Y, S:N501T, S:N501S). This residue is within the receptor-binding domain (RDB), that is important for both binding to ACE2 and for antibody recognition. A variant with S:N501Y, B.1.1.7 (also known as 20B/501Y.V1), was announced in the South East of England on December 14, 2020 [14]. Variants from this particular lineage are associated with multiple amino acid changes in the spike protein, including a deletion at 69/70 [16], Y144 deletion, and P681H (adjacent to the furin cleavage site). Rapidly spreading variants were also detected in South Africa. Viruses belonging to the lineage B.1.351 (also known as 20H/501Y.V2) were detected in December 2020 [17]. These variants are associated with multiple amino acid changes in spike protein, including S:N501Y, S:K417N, and S:D80A, but they do *not* have the deletion at 69/70 (Table 1). After the detection of these variants harboring similar genetic changes, genomic surveillance in countries experiencing high COVID-19 incidence started to report more variants with convergent genetic traits. In late 2020, a new variant was detected in Manaus, state of Amazonas, northern Brazil [18]. The new lineage, named P.1 (descendant of B.1.1.28, also known as 20J/501Y.V3), contains a unique constellation of lineage-defining mutations, including several amino acid changes of biological significance known as S:E484K, S:K417T, and S:N501Y. The P.1 lineage was identified in 42% (13 of 31) of RT-PCR positive samples collected between 15 and 23 December 2020, but was absent in 26 publicly available genome surveillance samples collected in Manaus between March and November 2020. These results indicate local transmission and possibly a recent increase in the frequency of a new lineage from the Amazon region [18]. Finally, two lineages originated in California, USA, have also emerged and increased in frequency from late 2020 to early 2021, named B.1.427 and B.1.429, both showing three amino acid substitutions: S:S13I, S:W152C, and S:L452R [19]. Variants from these two lineages have higher transmissibility (from 18.6 to 24%) when compared with wild type variants.

**Table 1:** Important variants of SARS-CoV-2 that emerged in late 2020

Lineage	Other designations	Likely origin	Key genetic changes
B.1.1.7	20I/501Y.V1	UK	69-70del, 144del, S:N501Y
B.1.351	20H/501Y.V2	South Africa	S:E484K, S:N501Y
P.1	20J/501Y.V3	Brazil	S:E484K, S:N501Y

Evidence from epidemiological and in vitro assays suggests that variants bearing the key genetic changes listed below are more transmissible

Some of the variants of concern have not only been associated with increased transmission potential but also with reduced susceptibility to neutralizing antibodies from convalescent patients and vaccines (immune scape) [20]. However, it is important to point out that cellular response conferred by vaccines is robust, identifies epitopes from many proteins beyond the Spike, and major losses of vaccine efficacy would mainly come as a cumulative effect of several widespread genetic changes that SARS-CoV-2 undergoes as it continues to spread [21]. Another important consequence of mutations is phenotypic changes of virulence. Initial studies have been suggesting that some variants may cause more severe illness, as already reported for B.1.1.7 [19, 22].

The emergence of SARS-CoV-2 variants with concerning phenotypes underscores the importance of genomic surveillance. The ability to track the spread of variants differs dramatically across regions, both in international and national levels [23, 24]. To prevent unnoticed viral spread, and to be able to promptly respond to new variants as they emerge, genomic surveillance needs to be incorporated as a routine activity by local departments of public health, sequencing a minimum percent of reported cases (1-5% of cases or more) per administrative division (towns, cities, counties, state, etc.).

## Human genetic susceptibility

Like in all infectious diseases, while pathogen genetics plays an important role, host genetics and physiology are key elements in determining the clinical course of disease in COVID-19 patients. The main unspecific symptoms of the disease are fever, myalgia, fatigue, and dry cough. As known, SARS-CoV-2 first affects the respiratory tract and then activates a systemic inflammatory response that can lead to interstitial pneumonia, up to more critical conditions. The worldwide infection fatality rate (IFR) is currently estimated around 2-3%, with a variability depending on different genetic and non-genetic factors, like sex and age above all [25]. It is a matter of fact that environmental factors contribute to the disease severity, but the health status represents a background that should not be underestimated.

### Comorbidities

Virus-host interaction plays a fundamental role in the disease’s outcome. Although most patients have a favorable prognosis, some groups are at higher risk, or “extremely vulnerable” to severe illness. These include first and foremost individuals with impaired immune system function; but also those with cancer, severe lung disease, such as chronic obstructive pulmonary disease (COPD), and pregnant women with cardiological disease [26, 27]. At “high risk” are also older patients, very obese individuals with obstructive sleep apnea syndrome (OSAS), and those with diabetes mellitus, neurological disorders, or heart, lung, liver, and kidney diseases, who are especially vulnerable to virus-induced acute respiratory distress syndrome (ARDS) [28]. This general risk stratification has exceptions: even young people without comorbidity may develop severe disease that may even become fatal. In order to explain this, several hypotheses have been formulated, including breakdown of immunological tolerance, the viral load, an innate immune inefficiency, and the presence of common or rare risk alleles in genes encoding proteins important for the biological cycle of the virus [29, 30]. A deeper knowledge of the involvement of the alterations affecting these pathways and the innate and adaptive immune system may represent a turning point for understanding the pathophysiological mechanisms of SARS-CoV-2 and the development of new therapeutic strategies. Variants in the genes that encode these proteins could contribute to different responses to infections.

### Genetic factors

Common and rare variants have been identified in different studies using different approaches (Table 2): Genome wide association studies (GWAS) and deep sequencing in selected cohorts and/or large biobank resources [35, 45–50]. Association studies made it possible to identify in a number of genes susceptibility alleles for severe disease phenotypes: however, so far, the risk values are too low (OR <2) to be regarded as predictive genomic markers (Table 2). Nonetheless, the additive effects of this low penetrance alleles might become important in the future through polygenic scores analysis [51]. On the other hands, highly penetrance alleles of genes encoding proteins involved in important pathways such as those of innate immunity (e.g., *TLR3*, *IRF7*) may be already useful for risk stratification and potentially useful for prognosis and treatment [30, 31]. A recent (not yet peer-reviewed) study that has appeared on medRxiv [52], did not find penetrating rare alleles associated with a severe disease phenotype in four different cohorts analyzed by whole-exome or whole-genome sequencing, thus questioning whether the data by Zhang et al. [31] have general validity. These discrepancies might be attributed at least in part to the heterogeneity of biobanks, to how phenotypic stratification is clinically assessed, and to how functional studies are conducted [31].

**Table 2:** Genetic risk factors for severe COVID-19

SARS-CoV-2 susceptibility gene variant or haplotype	Risk estimated [OR]	Frequency [MAF]	References
*TLR3*, *UNC93B1*, *TICAM1*, *TBK1*, *IRF3*, *IRF7*, *IFNAR1*, *IFNAR2* (autosomal-dominant model)	9	<0.001	Zhang et al. [31]
*IRF7*, *IFNAR1* (autosomal-recessive model)	>50	<0.001	Zhang et al. [31]
rs769208985—missense variant of *FURIN*	N.A.	<0.001	Latini et al. [32]
rs150892504—missense variant of *ERAP2*	N.A.	0.002	Hu et al. [33]
rs138763430—missense variant of *BRF2*	N.A.	0.002	Hu et al. [33]
rs147149459—missense variant of *ALOXE3*	N.A.	0.002	Hu et al. [33]
rs117665206—missense variant of *TMEM181*	N.A.	0.006	Hu et al. [33]
rs114363287—missense variant of *TMPRSS2*	N.A.	0.006	Latini et al. [32]
HLA DRB*27:07	N.A.	0.02	Novelli et al. [34]
rs74956615—3′UTR variant of *TYK2*	1.6	0.03	Pairo-Castineira et al. [35]
rs73064425—intronic variant of *LZTFL1*	2.1	0.08	Pairo-Castineira et al. [35], Ellinghaus et al. [36]
rs11385942—intronic variant of *LZTFL1*	1.8	0.07	Ellinghaus et al [36]
HLA DQB1*06:02	N.A.	0.08	Novelli et al. [34]
rs143334143—intronic variant of *CCHCR1*	1.9	0.09	Pairo-Castineira et al. [35]
HLA DRB1*15:01	N.A.	0.10	Novelli et al. [34]
rs6598045—5′UTR variant of *IFITM3*	N.A.	0.19	Kim et al. [37]
rs429358—missense variant of *APOE*	2.3-2.4	0.20	Kuo et al. [38]
rs9380142—3′UTR variant of *HLA-G*	13	0.29	Pairo-Castineira et al. [35]
rs2109069—intronic variant of *DPP9*	1.4	0.33	Pairo-Castineira et al. [35]
rs75603675—missense variant of *TMPRSS2*	N.A.	0.36	Latini et al. [32]
rs12329760—missense variant of *TMPRSS2*	0.9	0.39	Hou et al. [39]
rs657152—intronic variant of *ABO*	1.3	0.41	Ellinghaus et al. [36]
rs6020298—intronic variant of *TMEM189-UBE2V1*	1.2	0.58	Wang et al. [40]
rs10735079—intronic variant of *OAS1/3*	1.3	0.64	Pairo-Castineira et al. [35]
rs2236757—intronic variant of *IFNAR2*	1.3	0.71	Pairo-Castineira et al. [35]
rs3131294—intronic variant of *NOTCH4*	1.5	0.9	Pairo-Castineira et al. [35]
HLA B*46:01	2.1	N.A.	Lin et al. [41]
HLA-E*0101/0103	2.1/2.7	N.A.	Vietzen et al. [42]
*KLRC2*^del^	2.6/7.1	N.A.	Vietzen et al. [42]
HLA B*54:01	5.4	N.A.	Lin et al. [41]
c.2129_2132del, p.Gln710Argfs*18—frameshift variant of *TLR7*	N.A.	N.A.	van der Made et al. [43]
c.2383G>T, p.Val795Phe—missense variant of *TLR7*	N.A.	N.A.	van der Made et al. [43]
rs140312271—missense variant of *ACE2*	N.A.	N.A.	Novelli et al. [44]

*MAF* major allele frequency, *N.A.* not applicable, *OR* odds ratio

Identifying the role of rare variants is important in order to improve predictive testing, to unravel the pathogenetic mechanisms in different subgroups of SARS-CoV-2 positive subjects, and to develop personalized medicine for individual COVID-19 patients tailored to his or her genetic background. It is possible that, in a complex multifactorial and multigenic disease, such as COVID-19, several genetic and epigenetic factors are modulating the phenotypic manifestation, thus complicating the analysis of genotype-phenotype correlations. For example, it is known that non-coding RNAs (ncRNAs), and in particular microRNAs (miRNAs), are involved in the pathogenesis of SARS-CoV-2 infection and in host antiviral immune defense mechanisms [53]. Genes encoding miRNAs, like other genes, show inter-individual genetic variability, and several studies have shown that genetic variants in miRNA genes can, in some cases, affect their expression, maturation, and even affinity for their target genes [54, 55]. Thus, the high clinical variability of COVID-19 might be influenced by polymorphisms in microRNA target sites (MTS) or in miRNA sequences [55]. Genetic and epigenetic differences in miRNA expression in cells targeted by the virus during entry could affect the effectiveness of antiviral responses and therefore disease severity. Interestingly, it has recently been shown that level of expression of genes encoding proteins involved in virus attachment and entry (e.g., *ACE2*, *TMPRSS2*) varies with age and may provide a biological rationale for variability in presentation of COVID-19 [56]. Recently, Blume et al. [57] identified a new short isoform of ACE2 expressed in the airway epithelium. Short ACE2 is upregulated in response to interferon stimulation and rhinovirus, but not SARS-CoV-2 infection. Its expression is regulated independently of the primary transcript, with putative promoter elements identified upstream of the transcriptional initiation site of the short ACE2 transcript. The characterization of the functional elements of the ACE2 promoter and above all the factors involved in its regulation will help to understand better the mechanisms of pathology caused by SARS-CoV-2.

Newly the CHGE *Consortium* (Covid Human Genetic Effort, https://www.covidhge.com/about) initiated a study to enroll individuals (referred to as “resistant”) who were not infected with SARS-CoV-2 despite repeated exposure (e.g., care-givers or familiars of a patient with severe pneumonia), as evidenced by the absence of the disease and virus specific antibody titers in several tests. It is conceivable that these subjects carry monogenic variations that make them naturally resistant to virus entry, as previously shown for the *DARC* gene and *Plasmodium vivax*, *CCR5* and HIV, and *FUT2* and Norovirus [58–60]. Currently, there are no publications on this “resistant cluster,” but recently, Zeberg and Pääbo [61] have identified an haplotype on chromosome 12, which is associated with a ∼22% reduction in relative risk of becoming severely ill with COVID-19 when infected by SARS-CoV-2. Interestingly, this haplotype is inherited from Neanderthals and it is present at substantial frequencies in all regions of the world outside Africa. The genomic region where this haplotype occurs encodes proteins that are important during infections with RNA viruses.

## COVID-19 in Africa and in Latin America

Considering the multifactorial complexity of this disease, its rapid epidemic spread, and its fast evolving causative agent, COVID-19 is ravaging emerging countries, and its impacts in low-resource areas deserves our attention and action. We focus here on two regions of particular interest: Africa and Latin America.

On January 1, 2021, J. M. Maeda and J. N. Nkengasong published a paper on “The puzzle of the COVID-19 pandemic in Africa” [62]. The figure in the paper depicts the two COVID-19 “waves” seen in Europe are much less evident in Africa: rather, there has been a peak in July, 2020, followed by a gradual decrease and in turn by a gradual increase that is still on-going. The real “puzzle” is that a previous report (issued on March 26, 2020, by the WHO Collaborating Centre for Infectious Disease Modeling; MRC Centre for Global Infectious Disease) had predicted some 70 million cases and some 3 million deaths in Africa; in contrast, by November 22, 2020, the official figures were 2,070,953 cases and 49,728 deaths. The authors (who are at the Africa Centers for Disease Control and Prevention—the African CDC—in Addis Ababa, Ethiopia) mention “challenges with testing” as one possible reason for this striking discrepancy. This notion is also supported by a compilation from Nigeria [63]; according to official data, three-quarters of African COVID-19 cases are in South Africa and Egypt. An extreme case is that of Tanzania, where the COVID-19 epidemic is officially finished, and the last recorded case was on May 19, 2020. We think limited testing is a major reason for the paucity of cases in Africa.

Be that as it may, at least three factors of biological and epidemiological interest may have contributed to reducing the impact of the pandemic in Africa. First, in tropical Africa, sun exposure and UV radiation may inactivate an RNA virus rather more quickly. However, this hypothesis is not confirmed in other countries such as northern Brazil and other regions of the tropics where COVID-19 is found. Furthermore, the mode of transmission that occurs through droplets and aerosols does not fit this hypothesis. Second, the age distribution of the population is much younger. Third, a significant fraction of people (perhaps up to 30%) [64] may have antibodies against other corona viruses that cross-react with SARS-CoV-2 (even though only 2-8% have specific anti-SARS-CoV2 antibodies suggesting there may be, like in Europe, a large number of asymptomatic infections) [65]. Latin America has also been ravaged by COVID-19 and may be the world’s worst affected region [66]. Vaccinations against the virus are seen as the way forward and have begun (https://www.as-coa.org/articles/timeline-latin-americas-race-covid-19-vaccine) [67]. We must support a path to a world that defeats COVID-19 in all parts of the world. In fact international efforts by the Global Vaccine Alliance (GAVI), Coalition for Epidemic Preparedness Innovations (CEPI), Gates Foundations, WHO, etc. (https://www.weforum.org/agenda/2020/06/vaccines-immunization-poor-countries-coronavirus-covid-gavi) [68] are thankfully ramping up.

## New technologies accelerated into clinical practice

The pandemic has produced an unprecedented shift in the direction of basic and clinical research. We think it is remarkable that so many highly qualified research laboratories have been eager or willing to bring their experience in other areas to bear on this impelling worldwide problem, and we think it is commendable that they have had the courage to literally re-purpose their work. There has been also a deluge of publications: at March 30, 2021, the search item *COVID-19* in PubMed yields 118,065 papers; by the time this paper is published, there will be several thousands more publications. At the same time, new technologies for disease treatment and prevention are also being developed at unprecedented speed. In general, it takes a long time for advances to reach consumers, especially in the areas of biotechnology and health. However, the pandemic has led to a dramatic acceleration, including in the development of COVID-19 vaccines (Table 3) [69].

**Table 3:** Overview of the worldwide approved different types of COVID-19 vaccines

Vaccine	Product name	Vaccine type	Phase III efficacy	Doses	Storage	Prize per dose	Distribution
BioNTech/Pfizer	BNT162b2	mRNA	95%	2 doses (0.3 mL) [21 days apart]	−80 and −60 °C (−112 and −76 °F) for 6 months +2 and +8 °C (+36 and +46 °F) for 5 days	19.5 $	USA, EU, UK, Argentina, Australia, Bahrain, Canada, Chile, Costa Rica, Ecuador, Hong Kong, Iraq, Israel, Jordan, Kuwait, Malaysia, Mexico, Oman, Panama, Philippines, Qatar, Saudi Arabia, Singapore, South Korea, United Arab Emirates *COVAX* (*COVID-19 Vaccines Global Access*)
Moderna	mRNA-1273	mRNA	94.1%	2 doses (0.5 mL) [28 days apart]	−20 °C (−4 °F) for 4 months +2 and +8 °C (+36 and +46 °F) for 30 days	32-37 $	USA, EU, UK, Canada, Greenland, Iceland, Israel, Saudi Arabia, Singapore, Vietnam *COVAX* (*COVID-19 Vaccines Global Access*)
Oxford/AstraZeneca	ChAdOx1 AZD1222/Vaxzevria	Viral vector	81.3%	2 doses (0.5 mL) [10-12 weeks apart]	+2 and +8 °C (+36 and +46 °F)	1.5-4 $	USA, EU, UK, Australia, Bangladesh, Brazil, Canada, Greenland, India, Mexico, Nepal, Pakistan, Philippines, Sri Lanka, Taiwan, Vietnam *COVAX* (*COVID-19 Vaccines Global Access*)
Gamaleya (Sputnik V)	Sputnik V Gam-Covid-Vac	Viral vector	91.6%	2 doses (0.5 mL) [28 days apart]	−18 °C (0 °F) for 24 months +2 and +8 °C (+36 and +46 °F) for 3 months	< 10 $	Russia, India, Brazil, China, South Korea, Hungary, Argentina
Johnson&Johnson	JNJ-78436735 Ad26.COV2.S	Viral vector	72%	1 dose	−18 °C (0 °F) for 24 months +2 and +8 °C (+36 and +46 °F) for 3 months	10 $	USA, EU, Greenland, Canada *COVAX* (*COVID-19 Vaccines Global Access*)
Novavax	NVX-CoV2373	Virus-like particle	89%	2 doses [21 days apart]	−18 °C (0 °F) for 24 months +2 and +8 °C (+36 and +46 °F) for 3 months	16 $	USA, Canada
Sinopharm	BBIBP-CorV	Inactivated virus	78%	2 doses [3 weeks apart]	+2 and +8 °C (+36 and +46°F) Three years in storage	< 77 $	China, United Arab Emirates, Argentina, Bahrain, Egypt, Marocco, Pakistan, Perù
SinoVac	CoronaVac	Inactivated virus	50%	2 doses [3 weeks apart]	2-8 °C (36-46 °F) Three years in storage	14 $	China, Brazil, Turkey, Chile, Indonesia, Philippines
Covaxin (Bharat Biontech)	BBV152	Inactivated virus	81%	2 doses [21 days apart]	2-8 °C (36-46 °F) Three years in storage	1 $	India, Iran, Mauritius, Nepal, Zimbabwe

### Monoclonal antibodies

Although there are currently numerous studies to identify antivirals for SARS-CoV-2, the only compounds with therapeutic efficacy already in use are monoclonal antibodies (mAbs). mAbs are directed against the binding site of the SARS-CoV-2 spike protein receptor and they are able to block virus entry into human cells. mAbs have this name because they are produced by one type of immune cells (plasma cells) that are the progeny of a single parent cell [70]. mAbs, are laboratory-produced macromolecules engineered to bind to antigens of selected targets (e.g., cancer cells, microorganisms, viruses) [71]. The efficacy of mAbs has been successfully tested in other coronavirus infections (SARS-CoV) [72–74] and MERS [75]. During the past 12 months, several SARS-CoV-2 neutralizing mAbs have been isolated and characterized in several clinical studies (NCT04452318, NCT04497987). Some of these, such as bamlanivimab, casirivimab, and imdevimab, have been approved for emergency use in the treatment of mildly ill subjects. mAbs regarded as good candidates for clinical use have been derived by cloning B cells from patients who have recovered from COVID-19, or from other natural sources [76–82]. Bamlanivimab has been associated with a decrease in viral load and the frequency of hospitalizations or emergency department visits in outpatients with COVID-19 [83]; however, when it was co-administered with Remdesivir, it did not demonstrate efficacy among hospitalized patients who had COVID-19 without end-organ failure [84]. In a double-blind, phase I-III trial involving non-hospitalized COVID-19 patients, Weinreich et al. [85] investigated two fully neutralizing mAbs used in combination (casirivimab/imdevimab or REGN-COV2), in the aim to reduce the risk that treatment-resistant virus variants may emerge. REGN-COV2, developed by Regeneron Pharmaceuticals (USA), reduced viral load, with a greater effect in patients whose immune response to the virus had not yet been initiated, or who had a high viral replication at baseline. Very recent and unpublished data show that treatment with bamlanivimab (LY-CoV555) and etesevimab (LY-CoV016) together reduced the risk of hospitalization and death from COVID-19 by 70% (https://investor.lilly.com/news-releases/news-release-details/new-data-show-treatment-lillys-neutralizing-antibodies) [86]. Recently, EMA (European Medicines Agency) has completed review on the use of an additional mAb, regdanvimab (also known as CT-P59) to treat COVID-19 patients and concluded that regdanvimab can be used for the treatment of confirmed COVID-19 in adult patients who do not require supplemental oxygen therapy and who are at high risk of progressing to severe outcome (https://www.ema.europa.eu/en/news/ema-review-regdanvimab-covid-19-support-national-decisions-early-use) [87].

mAbs may be also useful for the prophylaxis of COVID-19 in persons who are at high-risk (health workers and first responders, pregnant women, ring-vaccination-type response to disease outbreak). By applying an innovative strategy, Miersch et al. [88, 89] have isolated high affinity synthetic mAbs from a phage library and thus developed a dynamic and rapid technological platform; this approach has the potential to identify in a short time mAbs against new virus variants. Synthetic engineering technologies may prove superior to natural cloning methods, as they offer exquisite control over the design of mAbs, that can prove more efficient. Similarly, Rappazzo et al. [90] identified rare broadly neutralizing antibodies (bnAbs) which can be engineered for improve neutralization potency and protection in vivo. Synthetic methods have the added advantage that they do not depend on natural repertoires, i.e., they are not limited by the need of accessing infected patients as a source of therapeutic agents. Andreano et al. [91] have isolated and characterized extremely potent neutralizing mAbs, suitable for prophylactic and therapeutic interventions of wild-type SARS-CoV-2 as well as emerging variants. Remarkably, an international research consortium recently developed a bispecific monoclonal antibody targeting two different SARS-CoV-2 sites, thereby preventing the virus from mutating to resist therapy. A single injection of the bispecific antibody provided protection against disease in mice. The antibody effectively reduced the viral load in the lungs and mitigated the typical COVID-19 inflammation [92].

### mRNA vaccines

Vaccines against SARS-CoV-2 infection were made in less than a year (Table 3). This research has undoubtedly been a great success of modern technology and public and private investments that have allowed to accelerate many of the processes required to develop a vaccine. But, of course, mRNA vaccines represent an epochal scientific and technical breakthrough.

mRNA was discovered in 1961 by Brenner et al. [93] and its ability to form drugs has been described already in 1989 [94, 95]. Since then, dozens of studies on the subject have been published, including a study on a vaccine against the MERS-CoV, which allowed the accelerated development of the SARS-CoV-2 vaccine [96]. This technology enables the synthetic production of the mRNA encoding any protein. mRNA vaccines have advantages over DNA vaccines. In general, lower doses are required to induce an immune response. Synthetic mRNA does not integrate into the cell’s genome, and no transcriptional step is involved, as the mRNA is directly translated, and then it undergoes degradation [97]. The vaccine activates both humoral immune system and cellular immune response, similar to live virus attenuated vaccine [98–100].

The safety data in adults on mRNA vaccines against COVID-19 have been reassuring. The most common side effects in adults are only local, within 7 days of receiving the first inoculation, and more common after the second dose [101, 102]. The side effects are mild-to-moderate; no severe-grade local side effects were reported [98, 103]. Systemic signs (fever of 38 °C) were reported in 75% of vaccine recipients. Subjects over the age of 65 suffered less systemic symptoms, but reported fatigue and headaches. During the rollout, some severe allergic reactions were also observed, but they were rare [104].

RNA-based vaccines efficacy, safety, and the potential for rapid, inexpensive, and scalable production makes them a powerful advantageous option to combat COVID-19, including probably new variants [105–110]. Widge et al. [111] have reported immunogenicity data 119 days after the first dose of vaccine (i.e., 90 days after the second dose) in 34 healthy adult participants who received two injections of 100 μg each. They provided further evidence that mRNA-1273, developed by National Institute of Allergy and Infectious Diseases (NIAID), the Biomedical Advanced Research and Development Authority (BARDA), and Moderna (ModeRNA Therapeutics, USA) have the potential to provide a durable humoral immunity, although for how long is of course not yet known.

Recently, a CDC study of about 4000 healthcare workers and workers belonging to essential categories were tested weekly for coronavirus after administration of the mRNA vaccine. The analyses found that people who had completed the vaccination course had a 90% reduced chance of becoming infected. Furthermore, the study showed that already from the first dose, the percentage after 14 days from the first injection was already close to 80%. Results clearly indicate the vaccine’s ability to interrupt viral transmission [112].

mRNA-based drugs are a promising novel platform that might be useful for the development of vaccines against emerging pandemic infectious diseases [113]. Furthermore, unconventional tools such as Cas13a-crRNA complex, an RNA-activated RNase, are being explored as novel therapeutics against SARS-CoV-2 infection, displaying promising results in reducing viral replication and symptoms in animal models [114]. RNA in human cells is susceptible to editing, and the RNA genome of SARS-CoV-2 is not an exemption. From a comprehensive sequence analysis of *Coronaviridae*, nucleotide changes have been identified that may be signatures of RNA editing by both ADAR deaminases and APOBEC deaminases. It has been suggested that this process may contribute to shaping the fate of both virus and patient [115].

## Conclusions

We are heartened by the progress in therapeutics and preventive strategies for COVID-19. Having achieved within less than 1 year from the start of the pandemic not only the development of a vaccine but also of several vaccines; having conducted the necessary clinical trials; having obtained approval from multiple regulatory agencies around the world; having actually already carried out mass vaccinations at least in some countries, is nothing short of spectacular [68]. While we can certainly not relent on the standard preventive measures (rigorous social distancing, frequent and thorough handwashing, avoiding poorly ventilated spaces, wearing of face masks as warranted), we are confident that the vaccines currently available and those in the pipeline will help defeat COVID-19 and allow a return to normality in due course [116, 117]. Improved therapies, such as mAbs, will also be useful in treating the disease. Understanding the virus entry and egress mechanisms could also open the door to promising therapeutic perspectives [118, 119]. Molecular testing and genomics will play a critical role in the detection of newly emerging variants.

Furthermore, we are mindful of our fellow humans in other countries, particularly in emerging economies. For many years, the Global Fund has provided funding for those affected by HIV; it has been a generous gesture, initially fueled by concerns that from a reservoir in Africa transmission elsewhere could take place. If HIV has been an epidemic, SARS-CoV-2 has sparked a pandemic, and it will be in everybody’s interest that it is controlled on a global scale because, as stated at the beginning of this paper, otherwise it will continue to affect the world. We are aware that WHO has launched the COVAX (COVID-19 Vaccines Global Access) scheme, and that the GAVI, backed by the Bill & Melinda Gates Foundation, the WHO, the World Bank, UNICEF, and others, has pledged $8.8 billion to reduce vaccine costs for poor countries. One possibility is that a good part of these funds will be absorbed by vaccinating persons from poor countries and provide immunization certificates to those who travel to richer countries, so that the latter will be protected. But we call on international organizations to set aside this potential narrow-sighted approach: and make instead vaccines available to all nations on a wide scale. We look forward to the WHO leading rigorously and expeditiously, lest their effectiveness is brought into question more than it has been hitherto. It is legitimate to hope that the lessons learned with COVID-19 will help in the future, not only in being more prepared for other similar emergencies but also in acting as one, for we have only one planet and only one humankind.

## Supplementary Information

**Additional file 1: Table S1.** List of genomes used in the analysis. We gratefully acknowledge the following Authors from the Originating laboratories responsible for obtaining the specimens, as well as the Submitting laboratories where the genome data were generated and shared via GISAID, on which this research is based.

## Data Availability

Data sharing is not applicable to this article as no datasets were generated or analyzed during the current study.

## Abbreviations

ARDS: Acute respiratory distress syndrome
ASEAN: Organization of Southeast Asian States
AU: African Union
BARDA: Biomedical Advanced Research and Development Authority
CDC: Centers for Disease Control
CEPI: Coalition for Epidemic Preparedness Innovations
CHGE: Covid Human Genetic Effort
COPD: Chronic obstructive pulmonary disease
COVAX: COVID-19 Vaccines Global Access
COVID-19: Coronavirus disease 2019
CTAP: Coronavirus Treatment Acceleration Program
EMA: European Medicine Agency
EU: European Union
EUA: Emergency Use Authorization
FAO: Food and Agriculture Organization
FDA: Food and Drug Administration
GAVI: Global Vaccine Alliance
GWAS: Genome Wide Association Studies
IFR: Infection fatality rate
mAbs: Monoclonal antibodies
miRNA: micro RNA
MERS: Middle East respiratory syndrome
NIAID: National Institute of Allergy and Infectious Diseases
OAS: Organization of American States
ORF: Open reading frame
OSAS: Obstructive sleep apnea syndrome
SARS-CoV-2: Severe acute respiratory syndrome coronavirus 2
UN: United Nations
WHO: World Health Organization
